# Raman and photoluminescence spectroscopy analysis of gamma irradiated human hair

**DOI:** 10.1038/s41598-021-86942-4

**Published:** 2021-04-12

**Authors:** Siok Ee Lam, Siti Nurasiah Mat Nawi, Siti Fairus Abdul Sani, Mayeen Uddin Khandaker, David Andrew Bradley

**Affiliations:** 1 Research Centre for Applied Physics and Radiation Technologies, School of Engineering and Technology, Sunway University, 47500 Bandar Sunway, Selangor Malaysia; 2 Department of Physics, University of Surrey, Guildford, Surrey GU2 7XH UK; 3 Department of Physics, University of Malaya, 50603 Kuala Lumpur, Malaysia

**Keywords:** Health care, Physics

## Abstract

Preliminary study has been made of black human hair, carbon concentration of some 53%, a model in examining the potential of hair of the human head in retrospective and emergency biodosimetry applications, also offering effective atomic number near to that of water. The hair samples were exposed to $^{60}$Co gamma rays, delivering doses from 0 to 200 Gy. Structural alterations were observed, use being made of Raman and photoluminescence (PL) spectroscopy. Most prominent among the features observed in the first-order Raman spectra are the D and G peaks, appearing at 1370 ${{\pm }} 18\,{\hbox {cm}}^{-1}$ and 1589 ${{\pm }} 11\,{\hbox {cm}}^{-1}$ respectively, the intensity ratio ${{{I}}}_{{{D}}}{{/}}{{{I}}}_{{{G}}}$ indicating dose-dependent defects generation and annealing of structural alterations. The wavelengths of the PL absorption and emission peaks are found to be centred at $592.3 \pm 12.5$ nm and $1077.4 \pm 7.3$ nm, respectively. The hair samples mean band gap energy (${{{E}}}_{{{g}}}$) post-irradiation was found to be $2.10 \pm 0.04$ eV, of the order of a semiconductor and approximately two times the ${{{E}}}_{{{g}}}$ of other carbon-rich materials reported via the same methodology.

Human hair fibre consist mainly of keratin proteins, the strands formed in a filamentous structure comprising three main morphological constituents: the cuticle (the outermost protective layer, formed by overlapping dead cells), the cortex (the thickest layer, formed of micro- and macrofibrils arranged parallel to each other, containing hard keratins related to increased disulfide crosslinks to provide strength), and in some cases, the medulla (the innermost central core of the hair, containing soft keratins to provide flexibility)^1,2^. Carbon is the major elemental constituent of human hair, on average accounting for some 50% of the mass, the rest being formed of oxygen (22%), nitrogen (16%), hydrogen (7%) and sulphur (5%)^3^. Strand diameters typically range from $\sim \ 30$ to $\sim \ 90 \upmu {\hbox {m}}^{2}$.

Numerous studies have made use of electron paramagnetic resonance (EPR) spectroscopy in studying the potential of human hair as a biological retrospective dosimeter and for emergency dose evaluation (in the dose range from several Gy up to tens of Gy). The focus of such applications has been on accidental exposure to ionizing radiation, focusing on stochastic effects and epidemiology, but also deterministic effects towards effecting rapid triage^4–7^. The origin of the EPR spectrum of human hair is mainly attributed to the melanin content, with associated free radicals generation resulting from irradiation, also mechanical damage to the hair^4,6^. The use of hair for EPR dosimetry has been reported to be greatly complicated, a result of a high background signal that confounds delineation of the radiation induced EPR signal, also because of time-dependent post-irradiation response variation. In particular, dose estimation has not been found possible at and beyond 120 h post-irradiation, the EPR signal being found to reduce by up to $\sim \ 95\%$^7,8^. Variation of EPR signal with hair colour and melanin type (brown to black eumelanins and yellow to reddish-brown pheomelanins^9^) has been well-noted, darker hair exhibiting greater EPR signal due to the greater concentration of melanin^6–8^. A further shortcoming of the EPR method includes that the EPR signal can be affected by the moisture content, no EPR signal being detected from wet hair^6^.

Raman spectroscopy provides the basis of a non-destructive and high-resolution technique, novel in terms of dosimetric utility, offering investigation of structural alterations as a consequence of extrinsic influences in carbon-rich materials, also providing valuable information on defects and crystallite size^10^. Moreover, Raman spectroscopy is extremely sensitive to short-, medium- and long-range order in solid carbon, which has rendered it a standard technique for characterisation of carbon materials^11^. Carbon, one of the most abundant elements in the Earth’s crust, exists in the form of three possible orbital hybridization types, ${\hbox {sp}}^{1}$ (linear geometry, formation of $\sigma$ and $\pi$ bonds), ${\hbox {sp}}^{2}$ (planar triangular, $\sigma$ and $\pi$ bonds) and ${\hbox {sp}}^{3}$ (tetrahedral, $\sigma$ bond), jointly responsible for the basis of organic compounds. Thus said, the various structures of carbon materials are mostly contributed to by ${\hbox {sp}}^{2}$ and ${\hbox {sp}}^{3}$ bonding^12^. In regard to Raman peak assignments for carbon materials it is noted that the first-order Raman spectrum exhibits a very strong in-plane stretching mode at 1581 cm$^{-1}$, the so-called graphite G peak, and a very strong broad band at $\sim$ 1360 cm$^{-1}$, denoted as disordered D peak in relation to defect concentration^11,13^. Prior dosimetric studies of carbon have focused on the use of non-biological carbon-rich materials, for applications within the radiotherapy dose regime, examining the structural alterations induced by sample formation and ionizing radiation^10,14,15^. Use has also previously been made of single-wall carbon nanotubes (SWCNT) buckypaper for thermoluminescence and X-ray photospectroscopy studies, providing analysis of the dose-dependent ${\hbox {sp}}^{2}$ to ${\hbox {sp}}^{3}$ hybridisation ratio of SWCNT^16–18^. Conversely, present work has investigated radiation effects in carbon-rich biological media, human hair specifically. Studies have concerned the structural characteristics of irradiated hair samples obtained from the head, in regard to the particular context the use of Raman and photoluminescence spectroscopy being considered novel. A further part of present work includes determination of the effective atomic number of hair, provided via use of elemental composition analysis of hair strands. The work is intended to gain greater fundamental understanding of structural alterations in hair samples, also examining the suitability of hair as a biodosimeter. Use is made of state-of-the-art technology, the overall intention being to seek an improved biological based radiation dose evaluation system for retrospective and emergency dosimetry in response to radiological accidents and incidents.

## Materials and methods

### Samples preparation, irradiation and characterisation

The black scalp hairs of a female donor were investigated in present work. For this preliminary study, ethical clearance and informed consent were not required since the donor is a member of present research group. The strands of hair (around 150) were cut into lengths of approximately 1 cm using a cleaned metal cutter and handled with stainless steel tweezer. The hair samples were first washed with acetone to provide for degreasing^19^ as well as to minimise the mechanically induced signal arising from cutting of the hair^20^, with subsequent washing with running water, and finally with double distilled water for cleaning of any possible external remnant contamination such as dirt and dust. The cleaned hair samples were then dried in the open air. Irradiations were made, delivering doses ranging from 0.5 to 200 Gy at a dose rate of 1.25 Gy/min using a $^{60}$Co gamma irradiator (mean energy 1.25 MeV). Also, the mass of hair sample was determined pre- and post-10 Gy irradiation, use being made of a Mettler Toledo electronic balance. The present work was carried out using facilities located at the Physics Department of the University of Malaya, including samples characterisation as described in the subsequent sections.

### Elemental analysis

Elemental compositions analysis of the unirradiated hair sample was conducted via use of Energy Dispersive X-ray (EDX) Spectroscopy, the cross-section of particular hair samples first being located using a Scanning Electron Microscope (SEM). SEM-EDX measurements were carried out using a Hitachi Tabletop SEM TM3030 (Japan), with $1000\times$ magnification, operating at an accelerating voltage of 15 kV. This provided the ability to identify the presence of the predominant elements in the hair, hydrogen being a particular exception due to its very low atomic number. Thus said, the natural presence of hydrogen in hair immaterially affects the effective atomic number, as will be appreciated below.

### Effective atomic number

For a particular multielemental medium, the effective atomic number ($Z_{eff}$) serves to characterise the overall energy absorption arising from the various photon-atom interaction processes. The simple power-law Mayneord formula (1) has been widely used for this purpose, incorporating an exponent of 2.94 in respect of the strongly atomic number dependent photoelectric effect. Accordingly, this relation defines the effective atomic number of a compound as follows^21^:

1 $$\begin{aligned} Z_{eff}=\ \root 2.94 \of {\sum {a_iZ^{2.94}_i}} \end{aligned}$$

with $a_i$ the relative electron fraction of the $i{\mathrm{th}}$ element with atomic number $Z_i$ in the 1-cm hair. Use has been made of the weight percentage of elements obtained from the EDX mapping for the calculation of $a_i$. The number of electrons per gram of an element of hair was calculated using the formula (2).

2 $$\begin{aligned} \frac{N_AZ}{A_w}\times W \end{aligned}$$

where $N_A$, *Z*, $A_w$ and *W* refer to respective Avogadro constant, atomic number, atomic weight and fractional weight of an element. The effective atomic number of a biological dosimeter close to that of water is preferred, water having similar radiation absorption and scattering properties to that of human soft tissue, thereby ensuring that the response is independent of the photon energy. Separately, an energy-dependent computation software, termed Auto-$Z_{eff}$ has been developed by Taylor et al.^22^, allowing rapid calculation (in 0.5–0.7 s) of effective atomic numbers for materials exposed to beam energies ranging from 10 keV to 10 GeV, again inputting the fractional mass of the elements of the material of interest. Auto-$Z_{eff}$ allows a user to define any material based on its elemental makeup, as for instance the hair used in present study. The Auto-$Z_{eff}$ software has also been employed in calculating the effective atomic numbers, both for mono-energetic gamma rays and also the entire gamma ray energy spectrum, accounting for both Compton as well as photoelectric interactions.

### Raman spectroscopy

**Figure 1:**
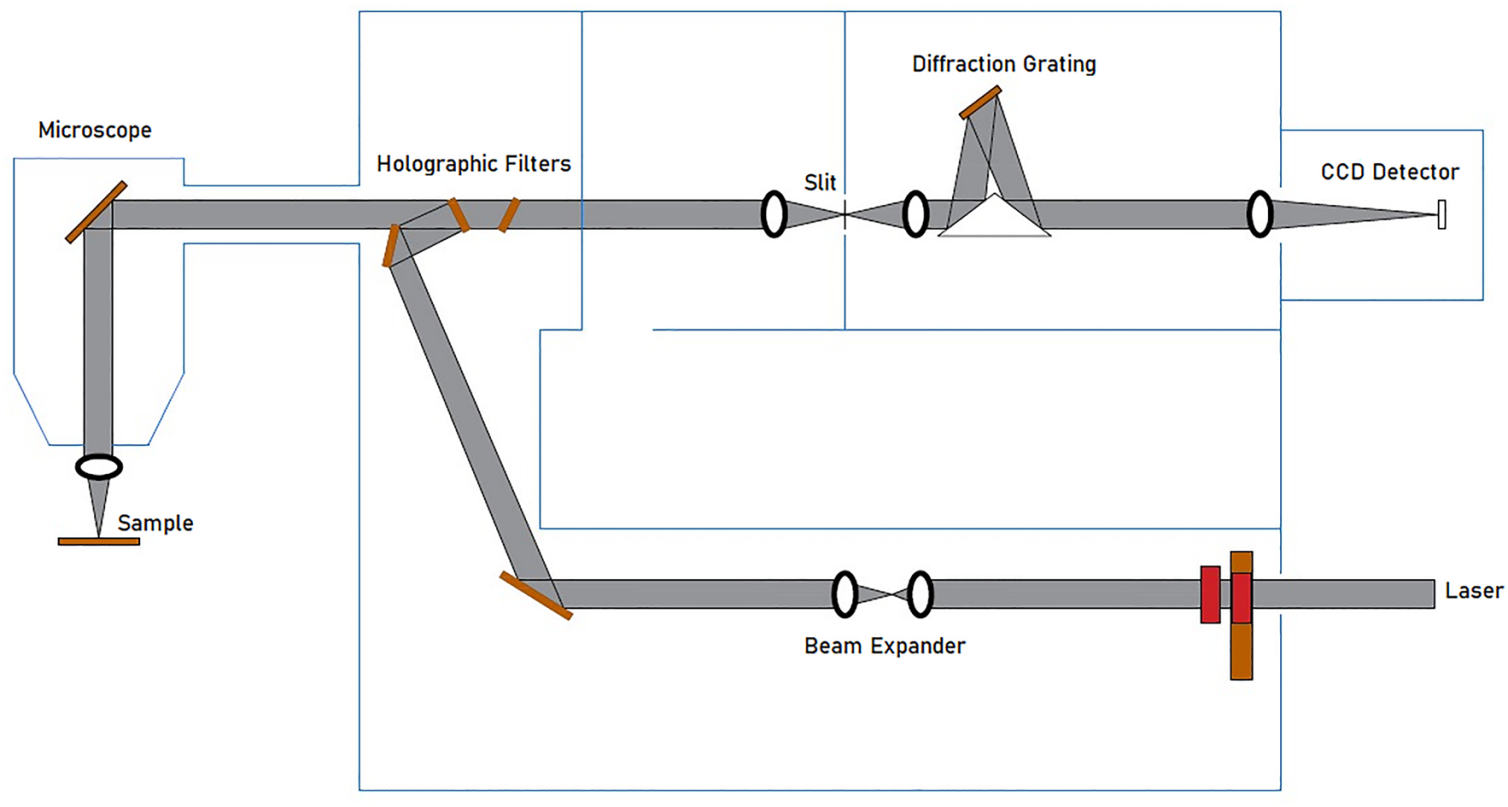
Schematic diagram of geometrical set-up of a Renishaw Raman system for Raman and PL measurements, adapted from literature^27,28^and drawn using AutoCAD LT 2021.

To provide for possible post-irradiation relaxation of the Raman signal, the Raman measurements were performed several days subsequent to the irradiation, a delayed situation envisaged in retrospective dosimetry. Use was made of a Renishaw inVia MicroRaman spectrometer (Fig. 1), with analysis carried out using a 532 nm excitation laser, the regions of interest being inspected using a $50\times$ objective lens^15^. The scattered light intensity, collected at $90^{\circ }$ to the Ar + laser excitation beam, was dispersed with a grating of 1800 lines/mm, detected using a Peltier-cooled charge-coupled device (CCD) camera of $578 \times 400$ pixels^15^. In order to determine the ratio of peak intensity at the D and G bands (${{\mathrm {I}}}_{{\mathrm {D}}}{\mathrm {/}}{{\mathrm {I}}}_{{\mathrm {G}}}$), ${{\mathrm {I}}}_{{\mathrm {D}}}$ and ${{\mathrm {I}}}_{{\mathrm {G}}}$ were obtained from deconvoluted Raman spectra, use being made of OriginPro 2018 software; smoothing and baseline correction have been applied on the raw Raman spectra prior to deconvolution.

The ratio ${{\mathrm {I}}}_{{\mathrm {D}}}{\mathrm {/}}{{\mathrm {I}}}_{{\mathrm {G}}}$ is known to depend strongly on the excitation laser energy^23^. Relating to this is use of the generalised Tuinstra–Koenig equation^24^, examining the relationship between crystallite size ${{\mathrm {L}}}_{{\mathrm {a}}}$ and ${{\mathrm {I}}}_{{\mathrm {D}}}{\mathrm {/}}{{\mathrm {I}}}_{{\mathrm {G}}}$, a relation widely adopted in characterising graphitic materials. An instance is the recent Raman spectroscopy study of graphitic samples by Abdul Sani et al.^14^, the Tuinstra–Koenig equation allowing use of any excitation laser energy in the visible range of the electromagnetic spectrum^23^. Herein, use has been made of the generalised formula (3) in order to determine the in-plane crystallite size ${{\mathrm {L}}}_{{\mathrm {a}}}$ of the carbon-based biomaterial, hair fibre in present circumstances, obtained by Raman spectroscopy using an excitation laser of wavelength ${\uplambda }$ of 532 nm (${{\mathrm {E}}}_{{\mathrm {laser}}}= 2.33$ eV).

3 $$\begin{aligned} {{\mathrm {L}}}_{{\mathrm {a}}}=(2.4 \times {{\mathrm {10}}}^{{\mathrm {-}}{\mathrm {10}}})\times {{\uplambda }}^{{\mathrm {4}}}{\times }{{(}{{{\mathrm {I}}}_{{\mathrm {D}}}}/{{{\mathrm {I}}}_{{\mathrm {G}}}}{)}}^{{\mathrm {-}}{\mathrm {1}}}. \end{aligned}$$

### Photoluminescence spectroscopy

Photoluminescence (PL) measurements were followed through using the same Renishaw inVia MicroRaman spectrometer (Fig. 1). Use has been made of the system with a $40\times$ objective lens and laser at 325 nm, providing for target excitation^14,26^. The excitation wavelength of 325 nm was used for PL measurements, experience showing that a choice of higher excitation wavelength would lead to reduction in PL intensity. The grating system offers 1200 lines/mm and the PL intensity was detected using a CCD camera of $578 \times 400$ pixels^14^. PL spectroscopy was used to acquire the band gap energy (${{\mathrm {E}}}_{{\mathrm {g}}}$) which is defined as the minimum energy needed to excite an electron from the valence band to the conduction band, and commonly used to categorise a material, according to whether it is considered an insulator, semiconductor or conductor. As such, use has been made of formula (4) to determine the band gap energy of hair, as follows:

4 $$\begin{aligned} {{\mathrm {E}}}_{{\mathrm {g}}}\ =\ {\mathrm {hc/}}{\uplambda } \end{aligned}$$

where ${\mathrm {h}}$, ${\mathrm {c}}$ and ${\uplambda }$ are the respective Planck’s constant, the speed of light and the corresponding wavelength of the absorption peak in PL spectra. For irradiated materials with undetermined electronic properties, the band gap energy has previously been used by Bradley et al.^26^, also considered to be useful in identifying semiconductor-like and semimetal biomaterials for biocompatible device fabrication for medical applications^25,26^.

## Results and discussion

### Elemental composition and effective atomic number

The mass of the 1 cm hair samples pre- and post-10 Gy irradiation were $0.077 \pm 0.006$ mg and $0.073 \pm 0.006$ mg respectively, no evidence being found within the level of precision for mass loss via degradation or denaturing processes. As indicated by EDX mapping, the abundance of carbon element in hair was found to be 53.43% (Table 1). Reflecting on prior dosimetric studies of carbon-rich media^14,26^, this finding would suggest the possible utility of hair as a passive dosimetric sensor of radiation, in particular for retrospective and emergency dosimetry. Here it can be noted that our prior work focused on carbon-rich pencil-lead graphite, a non-biological dosimeter, sometimes with in excess of 90% carbon. The results demonstrated great potential for the use of such graphite as thermoluminescent (TL) dosimeters in particular in monitoring dose delivery^15^. However, for present purposes it is clear that hair cannot be heated to very much more than several tens of degree Celsius without thermal damage occurring; as such we have discounted any use of TL analysis in current studies. Use of the well-established albeit photoelectric-biased Mayneord equation points to hair being a near water-equivalent biological dosimeter ($Z^{hair}_{eff}$ of 7.80, see Table 1) with a discrepancy of not more than 4% compared to the $Z^{water}_{eff}$ of 7.51$^{21}$. This has also been checked using the Auto-$Z_{eff}$ software that makes more comprehensive account of photon interactions, applying $^{60}$Co-spectrum-weighted incident radiation (very close to that of monoenergetic gamma 1.25 MeV, with a deviation of 0.0004%), obtaining a value of 6.79 for $Z^{hair}_{eff}$ (see Table 1). This shows a variation of just under 10% between Auto-$Z_{eff}$ value and the referenced value of 7.51. The robustness of the Auto-$Z_{eff}$ software has been supported; the calculated effective atomic number being found to be in good agreement with other methods exclusive of the Mayneord equation^29^. An uncertainty of 1–2% was reported for photon energies, spanning from 100 keV to 20 MeV, a region dominated by Compton and pair production interactions. Moreover, the spectrum-weighted $Z_{eff}$ calculated using Auto-$Z_{eff}$ for pencil-lead graphite (diameters 0.3–0.9 mm; $Z_{eff}$ 6.5–6.8)^15^ are found to be in the range of 6.14–6.20, a deviation of 6–9% compared to those of Mayneord-calculated $Z_{eff}$.

**Figure 2:**
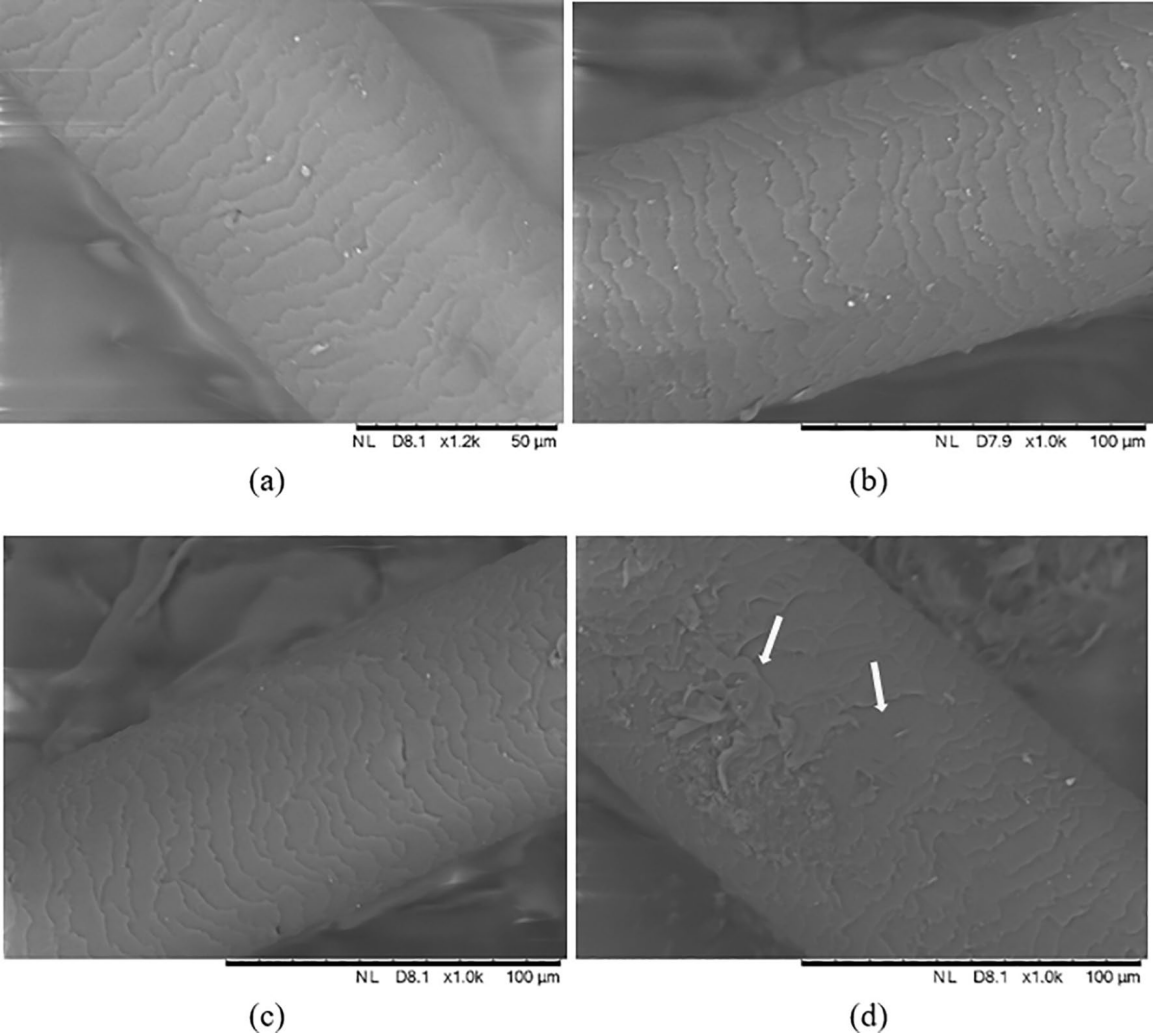
SEM images of the samples of hair, pertaining to: (**a**) non-irradiated hair and hair irradiated to doses of (**b**) 10 Gy, (**c**) 50 Gy and (**d**) 200 Gy. The arrows in (**d**) indicate irregularity of the cuticle scales pattern, seen at the highest dose level used herein.

The imbricate scales of hair cuticle (overlapping in similar way to roof tiles) can be clearly seen from the images obtained using SEM, as depicted in Fig. 2a–c. These indicate for hair irradiated in the dose range zero to 50 Gy that the features of normal hair are retained throughout, presumably with minimal moisture penetration or loss. This can be compared to porous hair characterised with raised cuticle scales, allowing greater moisture absorption but less moisture retention. Conversely, microstructural damage can be seen in the irregular cuticle scales pattern of the 200 Gy irradiation (Fig. 2d).

**Table 1:** $Z_{eff}$ calculations based on the average elemental compositions of single strand hair sample subjected to EDX mapping analysis.

Element	Weight (%)
C	$53.43 \pm 1.78$
O	$27.24 \pm 1.20$
N	$15.07 \pm 0.88$
S	$4.26 \pm 0.33$
$Z_{eff}$ (Mayneord)	7.80
$Z_{eff}$ (Auto-$Z_{eff}$)	6.79

### Raman spectroscopy analysis

**Figure 3:**
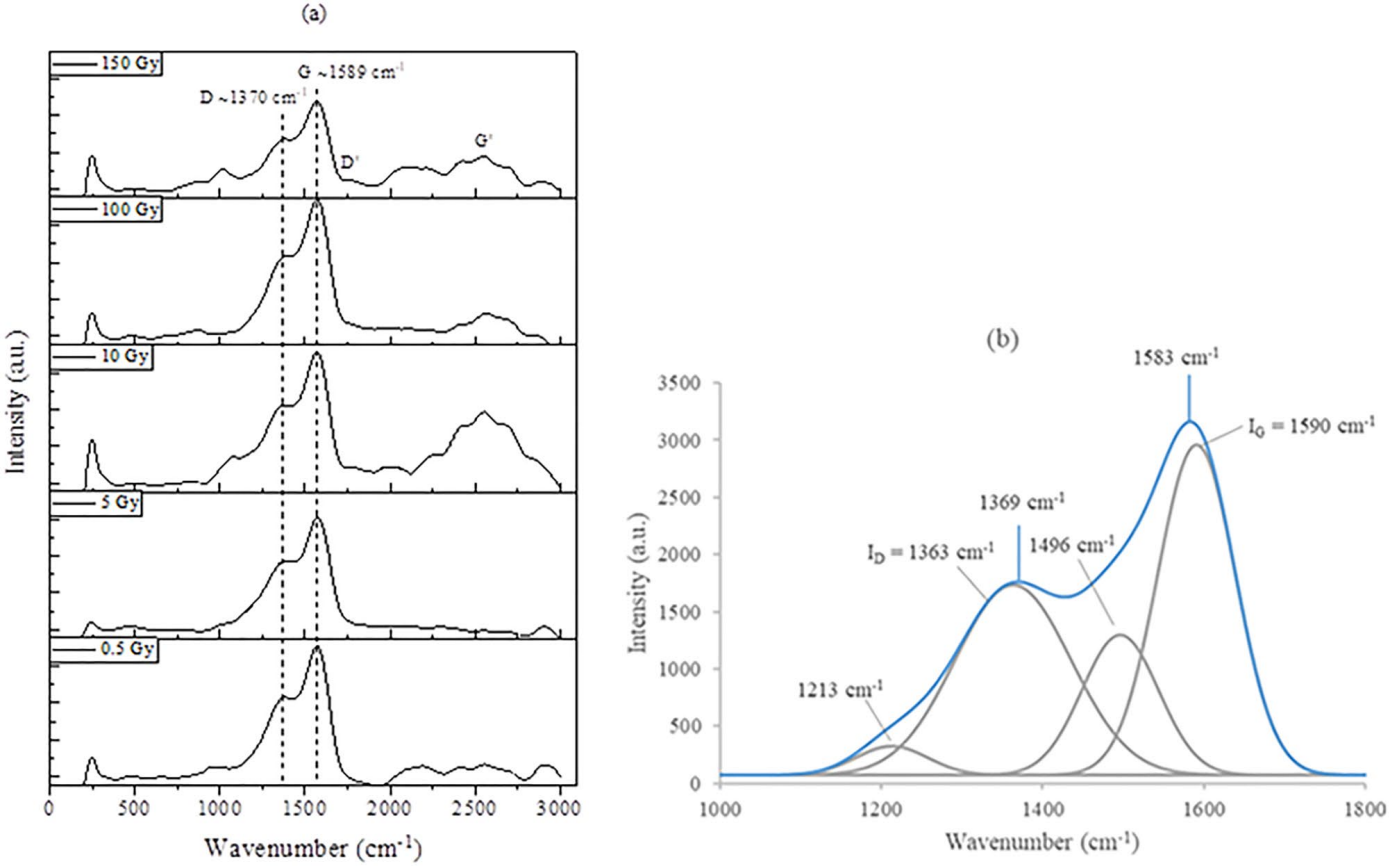
(**a**) First- and second-order Raman spectra of hair irradiated to various doses of $^{60}{\hbox {Co}}$ $\gamma$-rays. (**b**) Typical Raman spectra in a region extending from 1000 to $1800\,{{\hbox {cm}}}^{-1}$ for a dose of 0.5 Gy, revealing four deconvoluted peaks with their correspondingly labelled wavenumbers.

For the hair samples irradiated to doses from 0.5 to 200 Gy, wide Raman spectra in the range 0–$3000\,{{\hbox {cm}}}^{-1}$ were acquired using an excitation laser energy of 2.33 eV, as depicted in Fig. 3a. Focusing on the first-order Raman spectra (see Fig. 3b), deconvoluted Raman spectra in the range 1000–$1800\,{{\hbox {cm}}}^{-1}$ are observed to be dominated by two strong bands, with peaks at about $1370 {\pm } 18\,cm^{-1}$ and $1589 {\pm } 11\,{{\hbox {cm}}}^{-1}$ for doses from 0 to 200 Gy. These peaks are respectively the disorder induced D band (the so-called defect band) and the graphite G band. Note that the graphite G band arises from in-plane stretching of the hexagonal carbon rings or stretching vibration of ${\hbox {sp}}^{2}$ carbon (${\hbox {E}}{}_{2g}$ symmetry), while that for D band arises from in-plane breathing vibration of the aromatic ring structure (${\hbox {A}}{}_{1g}$ symmetry)^30^. An additional weak band at $\sim 1620\,{{\hbox {cm}}}^{-1}$ assigned as D’ is sometimes observed in the Raman spectra in disordered carbon, attributed to a splitting of the degenerate ${\hbox {E}}{}_{2g}$ first-order line^11^.

The broad peak of the second-order Raman spectra, ranging from $\sim 2500$ to $\sim 3000\,{{\hbox {cm}}}^{-1}$, is similar to that observed for human hair in another study^31^, being regarded as an overtone of the D band ($\sim 2700\,{{\hbox {cm}}}^{-1}$, sometimes known as 2D band) and an overtone of the combined D and G band ($\sim 2900\,{{\hbox {cm}}}^{-1}$) via second-order Raman scattering from irradiated hair^32,33^. Unlike the narrower ${\hbox {G}}'$ band at $\sim 2700\,{{\hbox {cm}}}^{-1}$ obtained from the Raman scattering of graphite materials in the study of Bradley and co-workers^10,14,15^, the absence of a well-defined G$^{\prime }$ band, as in Fig. 3a, is likely to be an indication of a second stage of amorphization, wherein the nanocrystalline graphite has progressively become amorphous carbon^34^. Regardless of the type of carbon-rich material, including the carboniferous hair studied herein, the two prominent bands with corresponding Raman shifts are very similar to the well-known D and G bands observed in the Raman spectra of disordered graphite^24^, and also in accordance with the reported ${{\mathrm {I}}}_{{\mathrm {D}}}$ and ${{\mathrm {I}}}_{{\mathrm {G}}}$ of a number of carbon-based studies (see Table 2).

**Table 2:** Raman D and G peak intensity of carbon-based materials found by several research groups.

Authors	Carbon materials	Dose (Gy)	${{{\mathrm {E}}}}_{{{\mathrm {laser}}}}$** (eV)**	${{{\mathrm {I}}}}_{{{\mathrm {D}}}}\,{(cm}^{-1}$)	${{{\mathrm {I}}}}_{{{\mathrm {G}}}}\,{(cm}^{-1}$)
Abdul Sani et al.^14^	Pencils H and 9B	0–20	2.41	$\sim$ 1348	$\sim$ 1578
Bradley et al.^10^	Mechanical pencil lead 2B & 8H	10	2.33	$\sim$ 1343	$\sim$ 1570
Pramanick et al.^31^	Pyrolyzed human hair	0	2.41	$\sim$ 1366	$\sim$ 1586
Qian et al.^35^	Pyrolyzed human hair	0	Not available	$\sim$ 1320	$\sim$ 1590
Huang et al.^36^	Human hair	0	1.58 and 1.96	$\sim$ 1375	$\sim$ 1580

**Figure 4:**
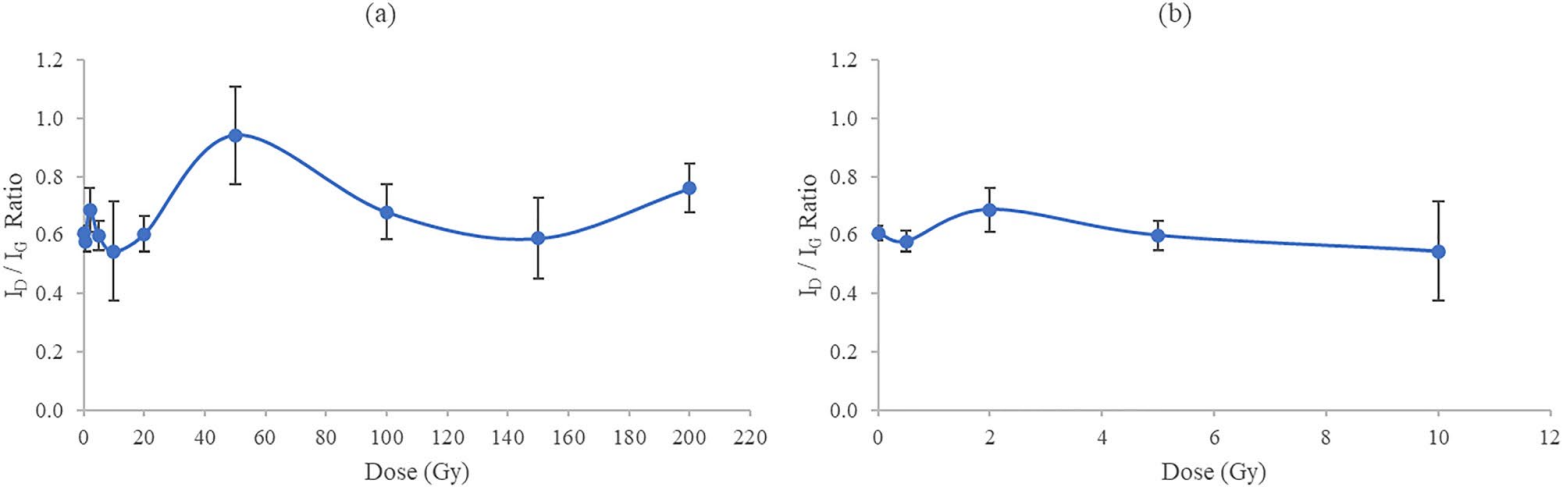
(**a**) The Raman intensity ratio ${{\mathrm {I}}}_{{\mathrm {D}}}{\mathrm {/}}{{\mathrm {I}}}_{{\mathrm {G}}}$ for hair samples irradiated using $^{60}{\hbox {Co}}\,\gamma$-rays, irradiation dose covering the range 0–200 Gy. (**b**) An expanded view of ${{\mathrm {I}}}_{{\mathrm {D}}}{\mathrm {/}}{{\mathrm {I}}}_{{\mathrm {G}}}$ for the dose range from 0 to 10 Gy.

The Raman intensity ratio ${{\mathrm {I}}}_{{\mathrm {D}}}{\mathrm {/}}{{\mathrm {I}}}_{{\mathrm {G}}}$ informs about the degree of structural disorder with respect to the perfect structure of graphite. Figure 4a shows the Raman intensity ratio ${{\mathrm {I}}}_{{\mathrm {D}}}{\mathrm {/}}{{\mathrm {I}}}_{{\mathrm {G}}}$ (referring to the ratio of peak heights) as a function of irradiation dose in the range 0–200 Gy and an expanded view depicting the intensity ratio within the lower dose range 0–10 Gy (Fig. 4b). The fluctuating behaviour in the ratio ${{\mathrm {I}}}_{{\mathrm {D}}}{\mathrm {/}}{{\mathrm {I}}}_{{\mathrm {G}}}$ (Fig. 4) is associated with the synchronous vibrational movement of carbon atoms, representing the onset of crystalline-amorphous transitions, depending upon several features such as ${\hbox {sp}}^{2}$ phase clustering, bond disorder, presence of ${\hbox {sp}}^{2}$ rings or chains and the ${\hbox {sp}}^{2}$/${\hbox {sp}}^{3}$ (graphite-like/diamond-like) ratio^34^. Raman spectroscopy in the visible light spectrum, as used in present study, is highly sensitive to the ordering of ${\hbox {sp}}^{2}$ sites in comparison to ${\hbox {sp}}^{3}$ sites as the visible photons excite only their $\pi$ states; direct probe of both the ${\hbox {sp}}^{2}$ and ${\hbox {sp}}^{3}$ sites can only be obtained using Raman spectroscopy in the ultraviolet spectrum with higher photon energy ($\sim$ 5 eV) wherein both $\pi$ and $\sigma$ states are being excited^34^.

The rise and fall in the ratio ${{\mathrm {I}}}_{{\mathrm {D}}}{\mathrm {/}}{{\mathrm {I}}}_{{\mathrm {G}}}$ as a function of dose is clearly seen across the entire range of investigated dose (Fig. 4a,b), a phenomenon previously found in a number of independent investigations by Bradley and co-workers^10,14,15^ for graphite-based materials. The disorder and subsequent partial recovery have been suggested to result from damage and subsequent dose-driven annealing of the defect density, with interchange between the dominance of defects (with ${{\mathrm {I}}}_{{\mathrm {D}}}$ increasing with respect to ${{\mathrm {I}}}_{{\mathrm {G}}}$) and defects annealing (with ${{\mathrm {I}}}_{{\mathrm {D}}}$ reducing with respect to ${{\mathrm {I}}}_{{\mathrm {G}}}$)^10^. Here, values of the intensity ratios ${{\mathrm {I}}}_{{\mathrm {D}}}{\mathrm {/}}{{\mathrm {I}}}_{{\mathrm {G}}}$ are found to range between $\sim$ 0.5 and $\sim$ 0.9 (see Fig. 4a); the lower values of ${{\mathrm {I}}}_{{\mathrm {D}}}{\mathrm {/}}{{\mathrm {I}}}_{{\mathrm {G}}}$ may be associated with reduced heteroatom (O, N and S) content^35^, likely due in part to internal heating of the hair during the course of irradiation. Note that most of the heteroatom content can be reduced via pyrolysis with increasing carbonisation temperature, leading to greater carbon content^31,35^. Also, the ${{\mathrm {I}}}_{{\mathrm {D}}}{\mathrm {/}}{{\mathrm {I}}}_{{\mathrm {G}}}$ of $\sim$ 0.9 obtained at 50 Gy is close to that of human hair-derived carbon fibre (${{\mathrm {I}}}_{{\mathrm {D}}}{\mathrm {/}}{{\mathrm {I}}}_{{\mathrm {G}}} = 0.99$)^31^ and human hair-derived carbon flake (${{\mathrm {I}}}_{{\mathrm {D}}}{\mathrm {/}}{{\mathrm {I}}}_{{\mathrm {G}}} = 1.10$)^35^, indicating that the structure is mostly glassy carbon.

**Figure 5:**
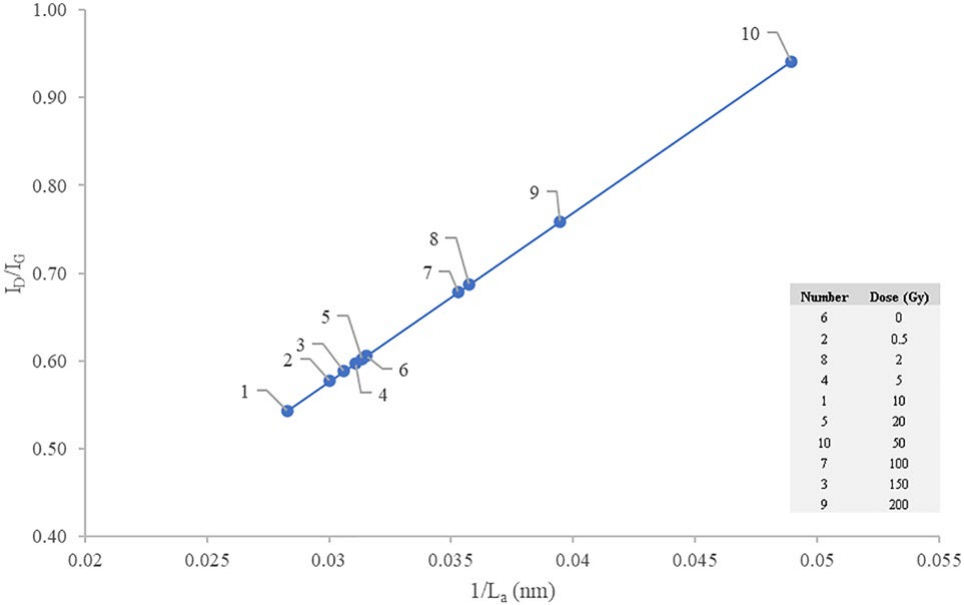
The intensity ratio ${{\mathrm {I}}}_{{\mathrm {D}}}{\mathrm {/}}{{\mathrm {I}}}_{{\mathrm {G}}}$ against $1/{{\mathrm {L}}}_{{\mathrm {a}}}$, ${{\mathrm {I}}}_{{\mathrm {D}}}{\mathrm {/}}{{\mathrm {I}}}_{{\mathrm {G}}}$ corresponding to $^{60}$Co $\gamma$-ray doses from 0 to 200 Gy. The inset shows the labelled points corresponding to the given doses.

Figure 5 shows the Raman intensity ratio ${{\mathrm {I}}}_{{\mathrm {D}}}{\mathrm {/}}{{\mathrm {I}}}_{{\mathrm {G}}}$ to be in direct proportion with 1/${{\mathrm {L}}}_{{\mathrm {a}}}$, with ${{\mathrm {L}}}_{{\mathrm {a}}}$ the crystallite size, clear indication that the greater the concentration of defects within the crystalline structure of hair, the smaller the hair carbon crystallite size, a phenomenon also seen in the study of graphite-rich pencils^14^. The smallest value of ${{\mathrm {L}}}_{{\mathrm {a}}}$, as in Fig. 5, was attributed to the maximum defect density obtained at a dose of 50 Gy (see Fig. 4a), representing an increase in disordered carbon.

### Photoluminescence spectroscopy analysis

**Figure 6:**
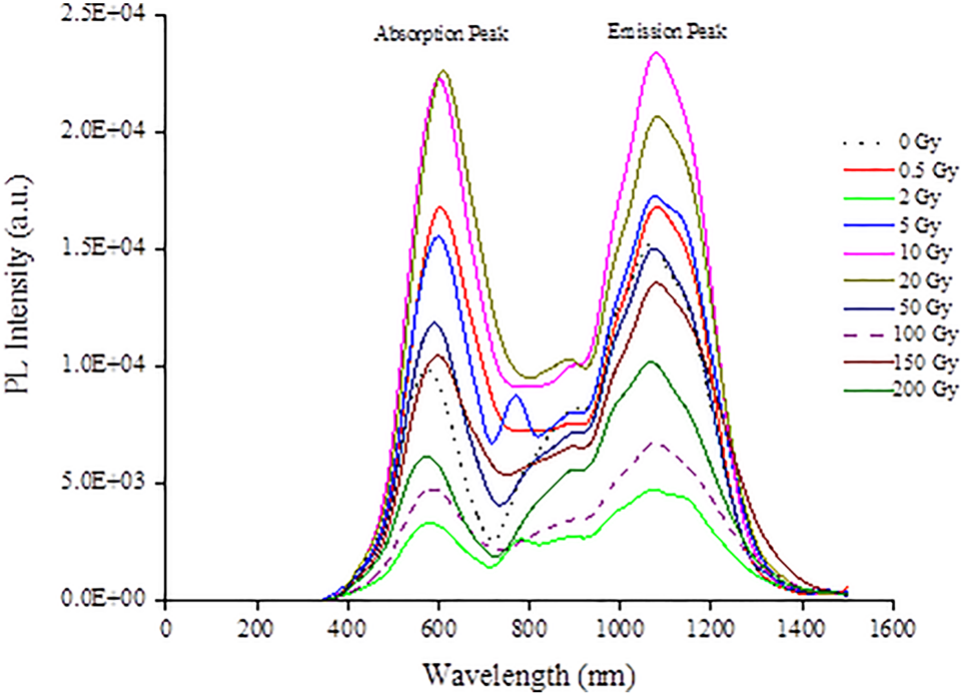
PL spectra of hair samples subjected to $^{60}{\hbox {Co}}$ gamma irradiation doses ranging from 0 to 200 Gy. The fluctuation in the values in both the absorption and emission amplitudes are suggested to arise from the radiation-driven suppression processes, including cross-linking and defects generation, giving rise to retrapping.

Hair, a carbon-based biomaterial, with around 53% carbon content exhibits two prominent PL peaks as seen in Fig. 6, corresponding to the absorption and emission peaks. The absorption peak corresponds to the absorption of laser photons with sufficient energy for photoexcitation whereas the emission peak corresponds to photoemission arising from electron–hole recombination. The wavelengths of the absorption and emission peaks are centred at $592.3 \pm 12.5$ nm and $1077.4 \pm 7.3$ nm, respectively, being also in accord with results for the graphite-based materials studies^14,26^. The loss of vibrational energy from that of the absorbance spectra as reported herein results in the shift of emission spectra to longer wavelengths (lower energy), at approximately 1077 nm. Based upon the de Broglie relation (4), the post-irradiation mean band gap energy of hair samples was determined to be $2.10 \pm 0.04$ eV, which can be considered as a semiconductor and approximately two times the ${{\mathrm {E}}}_{{\mathrm {g}}}$ of the graphite-rich pencils, with values of some $1.1{\hbox { eV}}$^14,26^.

**Figure 7:**
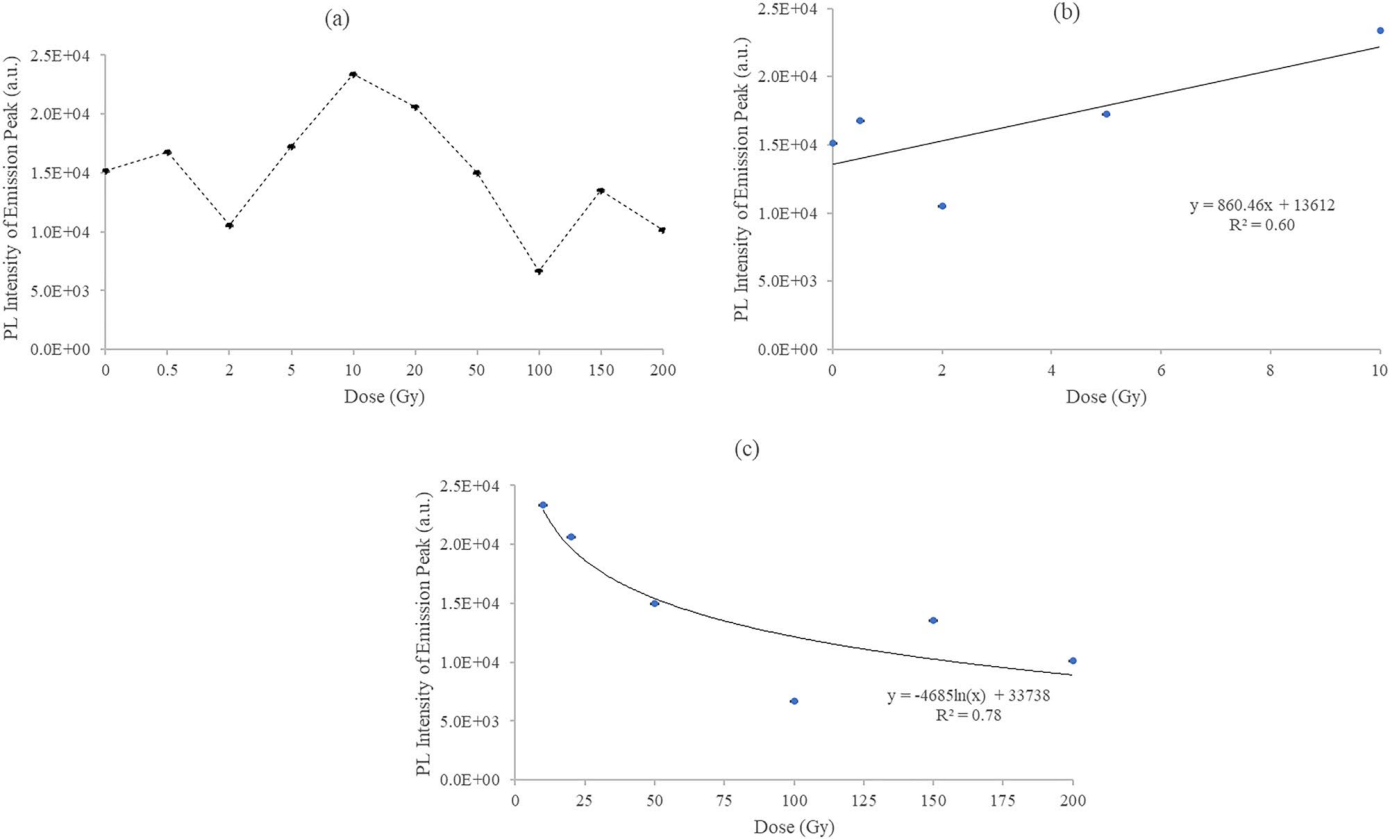
(**a**) Maximum PL intensity extracted from the emission peaks of PL spectra. The dashed line is a guide to the eye; (**b**, **c**) show the PL intensity of the emission peak plotted against the lower and higher dose regimes respectively. The error bars are one standard deviation values of the data points.

Figure 7a displays the maximum PL intensity extracted from the 1077 nm emission peaks of Fig. 6. Note the similarity in fluctuation of PL amplitude with dose as that seen in Fig. 4a, referring to the ${{\mathrm {I}}}_{{\mathrm {D}}}{\mathrm {/}}{{\mathrm {I}}}_{{\mathrm {G}}}$ ratio obtained from Raman spectra of the irradiated hair, in particular in terms of defect generation and suppression in the dose range from a few Gy to some 100 Gy, and from 100 to 200 Gy. A quasi linear association is found between PL intensity and $^{60}{\hbox {Co}}$ exposures (mean energy 1.25 MeV; linear energy transfer (LET) 0.20 $keV\,\upmu {{\hbox {m}}}^-1$), with doses from 0 to 10 Gy (${\hbox {R}}^{2}= 60\%$), measured at the 1077 nm emission peak (Fig. 7b). The likelihood is that the photoluminescent signals arise from the tryptophan content of hair, this being the fluorescent amino acid constituents of proteins^37^. Of note is that the tryptophan concentration in hair varies with age (being greater in first few years of life, 1–5 years, and highest in aging subjects ${\ge }$ 61 years), sex (higher in males), and hair colour (highest in white and grey hair, followed by black and brown hair)^38^. Figure 7c shows the dose-response curve for doses of 10 Gy and beyond, fitted to a logarithmic function, in which the photoluminescence from the hair decreases with increasing dose (${\hbox {R}}^{2}= 78\%$), evidence of hair damage being associated with the reaction of free radicals and direct deposition of energy^39^. Conversely to the situation presented for $^{60}{\hbox {Co}}$ irradiations for doses from 0 to 10 Gy, a downward trend in hair fluorescence for the same dose range was observed^39^, delivered in this case by $^{137}{\hbox {Cs}}$ (662 keV; LET $0.37\,keV\,\upmu {{\hbox {m}}}^{-1}$), obtained at an emission wavelength of 335 nm. The greater LET of the $^{137}{\hbox {Cs}}$ gamma rays is suggested to result in a greater degree of damage. The prediction of a flat dose-response at high gamma radiation doses^39^ of up to 200 Gy is not found in present work, the tryptophan being incompletely damaged or depleted. The damage due to gamma irradiation has been reported to be significantly reduced in dehydrated hair with 10–30% of water content relative to other biological samples such as viable epidermis of the skin with 70% water content^39–41^.

## Conclusion

Human hair is a readily available biological material, making it of potential interest as a bioindicator of dose evaluation in the particular circumstance of a sizeable radiological accident occurring. Based on prior evidence of the radiation sensitivity of carbon-rich media, human hair forming a potential example system, use has been made herein of a number of state-of-the-art analytic techniques, investigating the radiation sensitivity of human head hair. Interest is in whether such a system could enable rapid medical triage, informed by an estimate of dose. Strands of human hair were investigated via use of Raman and PL spectroscopy techniques, investigating the effects of $^{60}$Co gamma irradiation, for doses in the low dose regime up to 10 Gy and, beyond that, up to 200 Gy. Significant structural modifications have been observed at the given doses, manifest changes occurring in the shape and intensity of Raman spectra. PL measurements give rise to prominent absorption and emission peaks, the resulting mean ${{\mathrm {E}}}_{{\mathrm {g}}}$ being found to be $2.10 \pm 0.04$ eV, according with semiconductor-like behaviour. From these preliminary studies, it is evident that more detailed Raman and PL spectroscopy investigations are needed to justify that hairs could enable rapid medical triage, informed by dose estimation. Moreover, an additional study by infrared spectroscopy is necessary in order to highlight the influence of the gamma irradiation on the vibrational modes of the  C–C, C=C, C=O/C–O and C–S bonds. These would require study of a larger number of human hair samples, taking into account influencing factors such as location along the length of strands of hair measured with respect to the scalp, age, gender and natural hair colour, gaining further insight into dose-dependent structural alterations in human hair.
